# Bounding Box-Guided Diffusion for Synthesizing Industrial Images and Segmentation Maps

**DOI:** 10.3390/jimaging12030132

**Published:** 2026-03-16

**Authors:** Emanuele Caruso, Francesco Pelosin, Alessandro Simoni, Oswald Lanz

**Affiliations:** 1 Department of Engineering, Free University of Bozen-Bolzano, 39100 Bozen-Bolzano, Italy; oswald.lanz@unibz.it; 2 Covision Lab Scarl, 39042 Brixen-Bressanone, Italy; francesco.pelosin@covisionlab.com (F.P.); alessandro.simoni@covisionlab.com (A.S.)

**Keywords:** diffusion models, synthetic data, industrial inspection, defect segmentation, bounding-box conditioning

## Abstract

Synthetic dataset generation in Computer Vision, particularly for industrial applications, is still underexplored. Industrial defect segmentation, for instance, requires highly accurate labels, yet acquiring such data is costly and time-consuming. To address this challenge, we propose a novel diffusion-based pipeline for generating high-fidelity industrial datasets with minimal supervision. Our approach conditions the diffusion model on enriched bounding-box representations to produce precise segmentation masks, ensuring realistic and accurately localized defect synthesis. Compared to existing layout-conditioned generative methods, our approach improves defect consistency and spatial accuracy. We introduce two quantitative metrics to evaluate the effectiveness of our method and assess its impact on a downstream segmentation task trained on real and synthetic data. Our results demonstrate that diffusion-based synthesis can bridge the gap between artificial and real-world industrial data, fostering more reliable and cost-efficient segmentation models.

## 1. Introduction

Dataset synthesis has gained significant importance in recent years, particularly within the Natural Language Processing (NLP) community, where we witnessed major improvements in both academic and industrial applications [1,2,3]. These methods have proven especially valuable in scenarios where collecting and annotating real-world data are expensive or impractical.

In contrast, dataset synthesis in Computer Vision remains an emerging field and its usage is still under study [4,5]. Its potential to reduce labeling costs and mitigate data scarcity constitutes an appealing property for the deep learning paradigm. Despite its potential, the field remains relatively underexplored compared to its NLP counterpart. This is particularly true in domains where acquiring precise labeled data is both costly and time-consuming, such as industrial inspection, medical imaging, and remote sensing. In these domains, even small inaccuracies in annotation can significantly impact model performance, making synthetic data generation a compelling alternative.

Most of the recent research in synthetic data for vision has focused on text-to-image generation [6,7,8], leveraging generative models to create realistic visuals from textual descriptions. While these advancements have paved the way for creative applications and content generation, their direct applicability to real-world industrial settings remains limited. Industrial datasets, in particular, suffer from challenges such as class imbalances, labeling inconsistencies and high quality standards. These issues necessitate the development of tailored synthesis techniques capable of generating high-fidelity data hopefully with minimal manual intervention.

A critical challenge is the automatic creation of industrial dataset samples, where balancing efficiency with accuracy is difficult. Fully automated synthesis risks generating unrealistic or irrelevant samples, reducing the utility of the data. On the other hand, manual supervision, while improving accuracy, is often infeasible due to time and cost constraints—especially when dealing with complex imaging systems that go beyond human perception such as infrared imaging [9]. Industrial defect segmentation exemplifies this challenge, as it demands highly precise annotations to train reliable models.

To address these limitations, we propose a novel pipeline for generating realistic synthetic samples with cheap supervision. Our approach leverages diffusion models conditioned on human-provided bounding boxes to produce precise segmentation masks. By doing so, we unlock the generation of high-quality industrial datasets while exploiting human domain expertise but with a significant reduction in the burden of manual annotation.

In industrial settings, while diffusion models have been employed for data synthesis in classification tasks [10], their application to diffusion-based semantic segmentation remains limited, particularly when precise defect localization is required.

**Figure 1:**
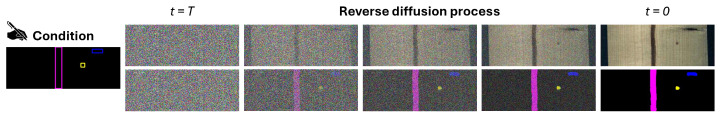
Overview of proposed diffusion-based approach that generates both RGB and segmentation map in industrial setting.

We present a diffusion-based approach, depicted in Figure 1, that generates RGB images and semantic maps leveraging an enriched bounding-box representation as conditioning. We compare it with a modified state-of-the-art approach on layout-conditioned generation [11]. Our baseline exhibits superior consistency in generating defects within the provided bounding-box annotations, making it preferable over existing generative pipelines. In this regard, we propose two metrics to quantitatively evaluate the obtained results. Ultimately, we provide some experiments showing the quality of the generated data by monitoring the performance of a downstream segmentation task trained on both real and synthetic data. Thus, we shed light on the potential of diffusion-based synthesis in bridging the gap between artificial and real-world industrial data, fostering more accurate and efficient Computer Vision models for segmentation.

To sum up, our main contributions are as follows:

- We introduce a novel synthetic data generation pipeline that leverages diffusion models conditioned on human-provided bounding boxes to generate high-fidelity industrial dataset samples.
- The proposed approach, thanks to an enriched bounding-box representation, ensures that the generated defects remain both realistic and accurately localized within the bounding-box boundary, enhancing segmentation consistency.
- By reducing the reliance on manual labeling, our method significantly lowers the cost and time required for curating industrial datasets while maintaining high annotation quality.
- We propose two metrics and evaluate our approach against a state-of-the-art conditioned diffusion pipeline, demonstrating competitive performance and improved control over defect placement.
- Our findings highlight the potential of diffusion-based dataset synthesis to improve industrial defect segmentation models, unlocking the development of more robust Computer Vision solutions in real-world settings.

## 2. Related Works

Synthetic data generation has been explored through various methodologies, each catering to specific domains and applications.

**3D Game Engines**. One prevalent approach leverages 3D game engines such as Unreal Engine [12], where meticulously crafted scenes or objects serve as high-fidelity proxies of reality. This method has been widely adopted, leading to the creation of extensive datasets and comprehensive frameworks [13,14,15], which have subsequently facilitated advancements in novel methodologies [16,17].

**GAN/Diffusion.** Another powerful paradigm involves neural generative models. Techniques such as GANs [18] and diffusion models [19] have demonstrated remarkable efficacy in producing high-fidelity synthetic data. These models have found widespread applications, ranging from medical imaging [20,21,22], self-driving car research [23,24], privacy preservation [25] and finally in robotics, where it has been investigated for pose estimation, as discussed in [26].

**Foundation Models.** Recently, foundation models have also been explored for synthetic data generation. Notably, COSMOs [27] facilitates the creation of entire synthetic video sequences, while large vision–text models have been widely utilized for generative applications [6,7,8].

**Layout-to-Image Generation.** Our pipeline generates RGB images with their corresponding labels starting from bounding-box conditions. One of the first methods of generation conditioned with a box-bound condition is Layout2Im [28], which uses word embeddings and latent vectors to encode the category and the appearance of the generated object.

After Layout2Im, many methods based on GAN architectures have been investigated for layout-to-image generation [29,30,31,32], but they suffer from the typical GAN limits such as unstable convergence. Many text-to-image diffusion models that are able to generate image conditioning over bounding boxes were investigated, such as GLIGEN [33], ControlNet [34], and InstanceDiffusion [35]. However, since our objective is defect synthesis within the industrial domain, large-scale text-pretrained models are neither necessary nor optimal for this task [36].

A related study [37] proposes a method for end-to-end RGB and label generation for satellite data. While their approach is purely generative, ours allows human intervention, granting users the flexibility to place annotations as needed. This distinction enhances the control and accuracy of label generation.

Thus, other recent methods for bounding-box-conditioned layout-to-image generation that do not rely on large-scale text-pretrained models are Layout Diffusion [11] and Stay Diffusion [36].

However, in this work we only compare the model with Layout Diffusion, as its official repository and implementation are publicly available [11], whereas to our knowledge, no public implementation for Stay Diffusion has been released, preventing direct experimental comparison.

## 3. Method

**Figure 2:**
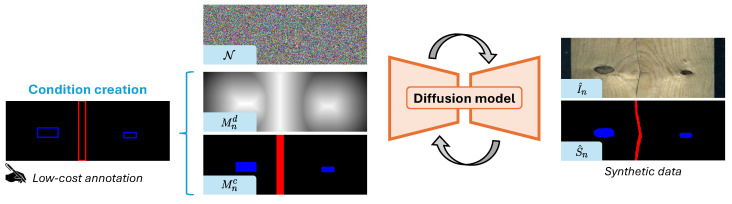
An overview of the proposed method: the user produces low-cost bounding-box annotations which are then converted into two representations (BASD and C-BASD). Later, these encodings are fed into the diffusion to condition the generation of both high-quality RGB and segmentation masks of wood defects.

In this section, we describe the proposed method shown in Figure 2.

### 3.1. Problem Statement

In this work, we address the challenging task of semantic segmentation in an industrial setting. Since the lack of annotated data is very common, a way to tackle this problem is to augment the annotations with synthetic samples. Thus, we aim to adapt a conditional diffusion-based pipeline to denoise both an RGB image and its segmentation map as an annotation.

Formally, we define a dataset $D=\{\left(I_{n},S_{n},B_{n}\right)|n=1,\ldots ,N\}$ where:

- $I_{n}^{H\times W\times 3}$ is an RGB image;
- $S_{n}^{H\times W}$ is the corresponding segmentation map composed of the discrete pixel values $c_{ij}\in \left\{1,2,3,\ldots ,C\right\}$ where *C* is the total number of classes;
- $B_{n}=\left\{b_{k}:\left(c,i_{min},j_{min},i_{max},j_{max}\right),k=1,\ldots ,K\right\}$ is a tuple that identifies the class of the object and its bounding-box location as the top left $\left(i_{min},j_{min}\right)$ and bottom right $\left(i_{max},j_{max}\right)$ corners.

Our method applies the diffusion process to the couple $\left(I_{n},S_{n}\right)$ conditioned on $B_{n}$. In the following section, we thoroughly describe how we preprocess the inputs and the training pipeline of the proposed method.

### 3.2. Data Preprocessing

The first step is to process the segmentation map $S_{n}$ and the bounding boxes $B_{n}$ to allow the diffusion process to work with continuous values.

**Segmentation map.** Since the goal is to generate synthetic samples according to the joint probability $p(I_{n},S_{n})$, we need to make sure that these data are in the same continuous space R. Drawing inspiration from [37,38], we convert the segmentation map into an analog bit representation. Formally, the pixelwise discrete segmentation values $c_{ij}$ are mapped into a binary code defined as

(1) $$\left\{1,2,3\ldots ,C\right\}\to {\left\{0,1\right\}}^{\left\lceil \log_{2}C\right\rceil }$$

After this encoding, the segmentation map dimension is $H\times W\times \lceil \log_{2}C\rceil$. As proven by previous works [38], this representation is more effective than one-hot encoding which is also less efficient in terms of the number of channels in the presence of a high number of classes *C*. After the binary encoding, a normalization is applied to change the range from [0,1] to [−1,1] which is the same as the RGB image $I_{n}$.

**Bounding box.** To condition the generation of the synthetic couple $\left({\hat{I}}_{n},{\hat{S}}_{n}\right)$ on the bounding boxes, we create an enriched representation of $B_{n}$ that encodes both spatial and class information. The spatial information is captured in terms of pixelwise encoding. Thus, we compute a Bounding Box-Aware Signed Distance (BASD) map $M_{n}^{d}$ that assigns to each pixel (i,j) the minimum distance to the nearest bounding-box boundary point. The distance value is positive inside a bounding box and negative outside. Moreover, a bounding-box class (C-BASD) map $M_{n}^{c}$ is computed accordingly, assigning to each positive value the corresponding class of the boundary point. We formally define the computation of $M_{n}^{d}$ and $M_{n}^{c}$ in Algorithm 1 and a visualization of the resulting maps can be seen in Figure 2.
**Algorithm 1** $M_{n}^{d}$ and $M_{n}^{c}$ computation.**Require:** Bounding boxes $B_{n}$**Ensure:** $M_{n}^{d}$ of size (H,W), $M_{n}^{c}$ of size (H,W)1:Initialize $M_{n}^{d}\leftarrow +\infty$ for all pixels $p_{ij}$2:Initialize $M_{n}^{c}\leftarrow 0$ for all pixels $p_{ij}$3:**for** each $b_{k}\in B_{n}$ with class *c* **do**4:      Compute boundary pixels of $b_{k}$:5:      $\beta \leftarrow \mathrm{Boundary}(b_{k})$6:      **for** each pixel $p_{ij}$ **do**7:             Compute distance to the closest boundary point:8:             $d_{\beta }\leftarrow \min_{(i_{\beta },j_{\beta })\in \beta }\sqrt{{\left(i-i_{\beta }\right)}^{2}+{\left(j-j_{\beta }\right)}^{2}}$9:             $d_{\beta }\leftarrow d_{\beta }*\mathrm{InOutSign}\left(p_{ij},b_{k}\right)$10:           Update $M_{n}^{d}$ and $M_{n}^{c}$:11:           **if** $|d_{\beta_{n}}\left|\;<\;\right|M_{n}^{d}\left(p_{ij}\right)|$ **then**12:                 $M_{n}^{d}\left(p_{ij}\right)\leftarrow d_{\beta }$13:                 $M_{n}^{c}\left(p_{ij}\right)\leftarrow c$14:            **end if**15:     **end for**16:**end for**

Before concatenating these two representation maps to the couple $\left(I_{n},S_{n}\right)$, the class map $M_{n}^{c}$ is encoded with the previously introduced analog bit paradigm obtaining an output dimension of $H\times W\times \lceil \log_{2}C\rceil$. Our encoding assigns a single class per pixel but still handles overlapping bounding boxes. When two boxes overlap, the class map forms a structured pattern reflecting the overlap location instead of arbitrarily selecting one class. Precisely, the overlap region is deterministically partitioned into two triangular subregions separated by the diagonal connecting opposite corners of the intersection area. The triangle containing the corner of the first bounding box is assigned to its corresponding class, while the opposite triangle containing the corner of the second bounding box is assigned to the second class. This allows the network to learn spatial relationships without needing explicit multi-label assignments, which a pure analog bit encoding cannot achieve.

### 3.3. Conditioned Diffusion Model

To synthesize realistic and structurally consistent images, we condition the denoising diffusion process on our enriched bounding-box representation. A UNet architecture takes as input $\left(x_{0},\;\left(M_{n}^{d},M_{n}^{c}\right)\right)$ where $x_{0}=\left(I_{n},S_{n}\right)$. The output is the couple $\left({\hat{I}}_{n},{\hat{S}}_{n}\right)$ comprising an RGB image plus its segmentation map with dimension $H\times W\times 3+\lceil \log_{2}C\rceil$.

Given a clean sample $x_{0}$, the forward diffusion process gradually adds Gaussian noise:

(2) $$q\left(x_{t}|x_{0}\right)=N\left(x_{t};\sqrt{\alpha_{t}}\;x_{0},\;\left(1-\alpha_{t}\right)I\right),$$

where $\alpha_{t}$ is the noise scheduling coefficient. The reverse process learns to reconstruct $x_{0}$ while incorporating the structural constraints from the conditioning $\left(M_{n}^{d},M_{n}^{c}\right)$:

(3) $$\begin{array}{cc} \; & p_{\theta }\left(x_{t-1}|x_{t},M_{n}^{d},M_{n}^{c}\right)=\\ \; & N\left(x_{t-1};\mu_{\theta }\left(x_{t},t,M_{n}^{d},M_{n}^{c}\right),\sigma_{t}^{2}I\right). \end{array}$$

where $\mu_{\theta }\left(x_{t},t,M_{n}^{d},M_{n}^{c}\right)$ is the predicted denoised estimate and $\sigma_{t}$ is the variance of the noise distribution.

The diffusion model is trained by minimizing the noise prediction loss:

(4) $$E_{x_{0},M_{n}^{d},M_{n}^{c},t,\epsilon }\left[∥\epsilon -\epsilon_{\theta }\left(x_{t},t,M_{n}^{d},M_{n}^{c}\right){∥}^{2}\right],$$

with ϵ∼N(0,I) representing the injected Gaussian noise. This formulation ensures that the generated samples adhere to both the semantic structure encoded in the segmentation and the spatial constraints provided as bounding-box conditioning.

## 4. Experiments

In this section, we discuss the implementation details and the industrial dataset we used for our experiments. Finally, a thorough comparison between our approach and a state-of-the-art conditional diffusion model [11] is assessed in terms of quality and consistency.

### 4.1. Experimental Setting

**Diffusion model.** The proposed method follows the DDPM [19] paradigm with a UNet [39] architecture trained from scratch. We modified the input and output channels accordingly to support our bounding-box encoding representation and the denoising of the segmentation map. Both during training and testing, the number of denoising iterations was set to 1000. We trained for 300 epochs using AdamW [40] as the optimizer with a learning rate of $1\times 10^{-5}$ and a batch size of 8.

**Downstream task.** For the semantic segmentation downstream task we employed a UNet architecture with a ResNet-18 [41] backbone. We used a single network for each segmentation class to avoid class balancing problems and concentrate on the synthetic data assessment. The training lasted for 100 epochs using AdamW as the optimizer with a learning rate of $1\times 10^{-5}$ and a batch size of 64 on a single Nvidia RTX 4090.

**Dataset.** Although several open industrial defect datasets are available, most present significant practical limitations when used as a single training source. The first group of datasets is constrained by limited scale [42,43]. Such limited data volume is insufficient for training diffusion models. The second group includes datasets with single-class or very low class diversity, such as concrete crack benchmarks [44], which provide high-quality masks but model only one defect category, thereby preventing evaluation of multi-class segmentation capability. The third group comprises datasets with strong class imbalance or scarce defect-positive samples, such as KolektorSDD/SDD2 [45,46], where only a small fraction of images contain annotated defects, limiting effective supervised learning of defect regions. Additionally, some classical benchmarks such as DAGM 2007 either rely partially on synthetic data or lack the variability and scale typically encountered in real industrial production.

In contrast, the Wood Defect Detection dataset [47] combines large-scale data volume, multiple defect categories, real production-line acquisition, and pixel-level annotations for all defect instances. It contains 20,276 images with semantic segmentation and bounding-box annotations of 10 different classes of wood defects. In our experiments, we decided to aggregate the 4 classes of knots and exclude the blue stain and overgrown classes that are underrepresented. Thus, we obtained a dataset comprising 20,107 images with a total of 5 defect classes (knot, crack, quartzite, resin, and marrow).

**Figure 3:**
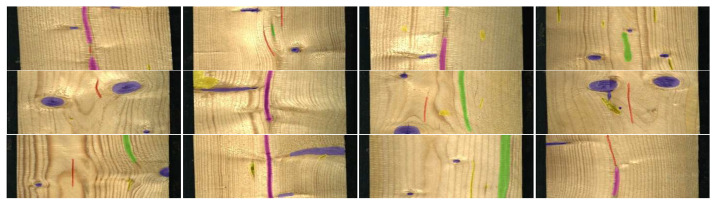
Some samples of the Wood Defect Detection [47] with the semantic segmentation labels. The wood defects are the following: knot (blue), crack (red), quartzite (green), resin (yellow), and marrow (magenta).

Moreover, we split the dataset into three subsets: 70% for training the diffusion model, 20% for training the segmentation model, and 10% as a fixed real test set. Additionally, the bounding-box annotations from the 20% real split are used to generate synthetic data for evaluating the semantic segmentation task. Figure 3 illustrates some samples from the original dataset.

### 4.2. Data Synthesis Assessment

To assess the quality of synthetic data, we compare our approach with the current state-of-the-art layout-conditional diffusion model [11], utilizing its original code implementation and adapting it to take non-squared images. Specifically, we focus on evaluating the consistency between the generated defects and their corresponding bounding-box constraints. To quantify this relationship, we introduce two metrics, the Segmentation Alignment Error (SAE) and the Empty Bounding-Box Rate (EBR).

**Segmentation Alignment Error (SAE).** With this measure, we quantify how many generated defect pixels fall outside their designated bounding boxes, indicating misalignment between the generated defects and their constraints. Formally, let:

- $\hat{P}$ be all the generated pixels of segmented defects;
- ${\hat{P}}_{out}$ be the generated pixels that fall outside the bounding boxes.

Thus, we define the metric as follows:

(5) $$\mathrm{SAE}=\frac{{\hat{P}}_{out}}{\hat{P}}$$

where a lower value indicates that the model is more consistent with the generation condition.

As shown in Table 1, the method proposed in [11] struggles to maintain defect placement within the bounding boxes, resulting in a very high mean SAE of 46.77% across all the defects. In contrast, our approach, leveraging a dual bounding-box encoding strategy (BASD and C-BASD), significantly improves alignment, with only 4.99% of generated pixels falling outside the given regions.

**Empty Bounding-Box Rate (EBR).** To assess whether the generated defects correctly fall within their designated bounding boxes, we define the Empty Bounding-Box Rate (EBR). This metric quantifies how many bounding boxes remain empty, meaning no synthetic pixels are generated inside them. Formally, let:

- $B_{all}=\left\{b_{k}\;|\;b_{k}\in B_{n},\;n=1,\ldots ,N\right\}$ be the set of all bounding boxes;
- $B_{miss}=\left\{b_{k}\;|\;b_{k}\in B_{all},\;G\cap b_{k}=\emptyset \right\}$ be the subset of bounding boxes that contain no generated pixels.

Thus, we define the metric as follows:

(6) $$\mathrm{EBR}=\frac{|B_{miss}|}{|B_{all}|}$$

where higher values indicate that a larger number of bounding boxes are missed during generation, signifying a poorer retrieval of the provided conditioning.

As reported in Table 2, the EBR metric shows the superiority of our proposal in retrieval abilities by a large margin. Specifically, our average EBR lies around 5.51% on the total amount of bounding boxes and surpasses the competitor by more than 20% points [11].

**Visual sample quality.** To further analyze the quality of the generated synthetic images, we report the Fréchet Inception Distance (FID) [48], the Kernel Inception Distance (KID) [49], and LPIPS [50]. FID and KID are computed between real and synthetic images using features extracted from the InceptionV3 network [51]. Specifically, we evaluate the statistics at different intermediate feature layers (corresponding to different spatial resolutions: 2048, 768, 192, and 64 channels), following standard practice to assess both high-level semantic alignment and lower-level texture fidelity.

Higher-level features (like the 2048-dimensional Inception embedding) capture broader semantic structures and distributional alignment, but are less sensitive to fine texture and local structural coherence than lower-level features. Therefore, a method that excels at texture/detail accuracy (which matters more for defect realism) can sometimes appear worse at the highest feature layer, because those layers emphasize global layout similarity rather than local perceptual fidelity.

As shown in Table 3, our method consistently improves FID and KID at lower-level feature representations, indicating better local structural fidelity. Moreover, LPIPS computed across multiple backbones (AlexNet, VGG-16, and SqueezeNet) confirms improved perceptual similarity and robustness across architectures. All metrics were computed using the same number of real and synthetic samples for both methods to ensure a fair comparison: FID and KID are computed using the full real test split (10% of the dataset, *N* images) and an equal number (*N*) of synthetic samples generated considering the same bounding-box annotations for each method. Each metric is evaluated over three independent sampling runs using the same trained model, and we report mean ± standard deviation.

**Figure 4:**
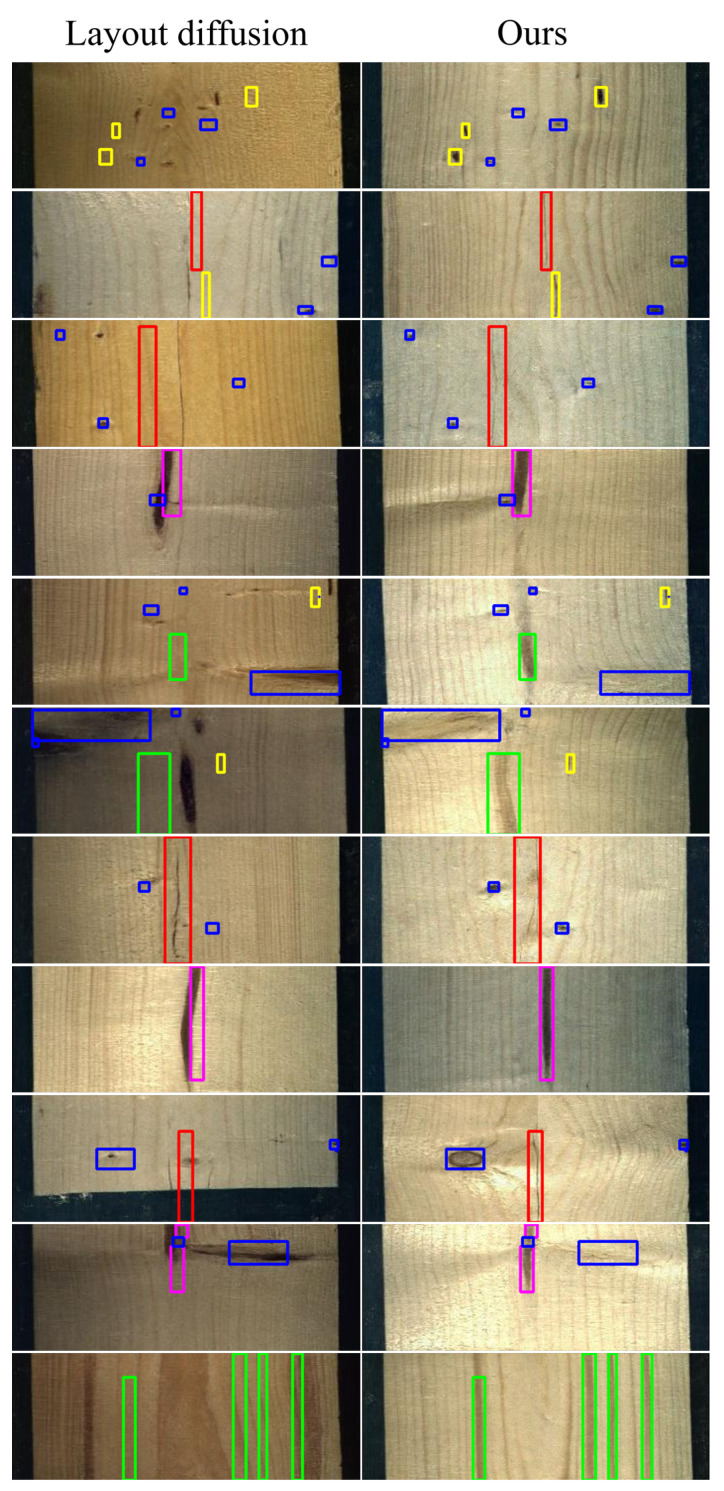
Qualitative comparison between our method and Layout Diffusion [11]. Each method shows the generated RGB image with respect to the bounding-box condition. The wood defects are the following: knot (blue), crack (red), quartzite (green), resin (yellow), and marrow (magenta).

**Figure 5:**
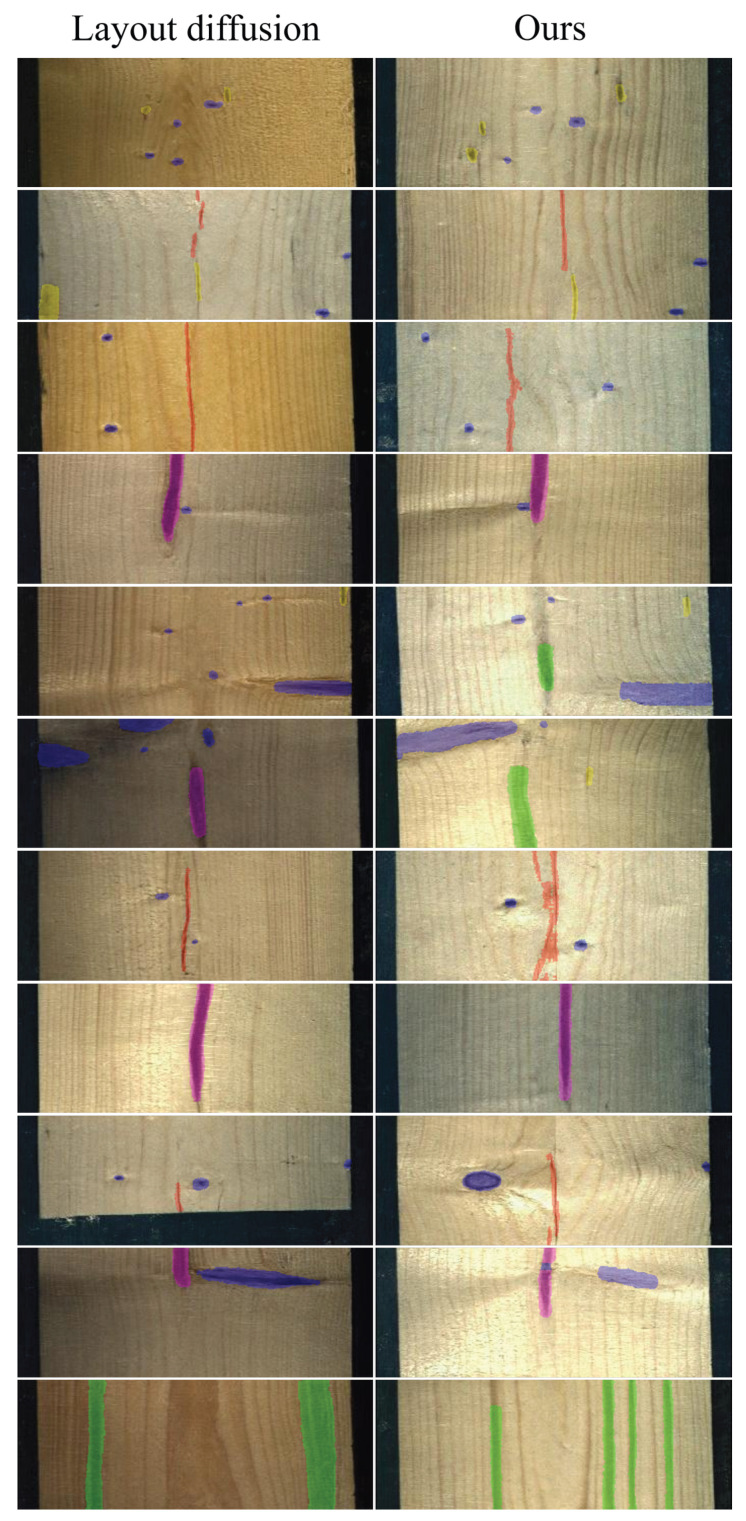
Qualitative comparison between our method and Layout Diffusion [11]. Each method shows the generated RGB image with respect to the defect segmentation map. The wood defects are the following: knot (blue), crack (red), quartzite (green), resin (yellow), and marrow (magenta).

**Qualitative results.** To further illustrate this comparison, Figure 4 and Figure 5 depict qualitative examples. Moreover, the results demonstrate that [11] not only fails to confine defects within the bounding boxes but also occasionally generates wrong segmentation labels.

### 4.3. Downstream Task Evaluation

To evaluate the effectiveness of our synthetic data, we conduct a semantic segmentation experiment using a UNet architecture trained on different data configurations.

Starting from the 20% split, we use the original bounding-box annotations as guidance to generate pairs of images and labels. We do so for both methods, ours and [11]. We then use this synthetic split to train the segmentation pipeline. Moreover, to ensure a fair comparison between approaches, we discard synthetic pixel labels generated outside the conditioning bounding boxes. This step is applied identically to all methods and does not modify the generated RGB images. Its purpose is to isolate the effect of bounding-box-guided supervision during downstream training, while global consistency and leakage are independently evaluated through SAE and EBR.

Table 4 presents the F1 scores computed on the 10% real test split, where we compare models trained on real data, synthetic data, and a combination of both. Notably, when training on synthetic data alone, our approach surpasses [11] by an impressive 10%, demonstrating its ability to generate more valid training samples. This highlights the superior quality and consistency of our synthetic segmentation maps, which provide a more reliable learning signal for the segmentation task.

When incorporating real data into the training process, the performance gap between the two methods narrows, as real samples provide a strong baseline. However, even in this hybrid setting, leveraging our synthetic data leads to the best overall F1 score, achieving a +1.17% improvement over using only real data. This behavior suggests that the diffusion model captures the real data distribution reasonably well, producing synthetic samples whose visual and statistical properties are close to those of the real data. As a result, most of the performance gain on segmentation is achieved when replacing real data with synthetic data, while adding synthetic data on top of real data yields diminishing but still measurable returns. This result highlights that our method complements real-world annotations and can achieve strong performance with fewer labeled samples, potentially reducing the time and effort required for manual labeling in industrial scenarios.

### 4.4. Ablation Study

To isolate the contribution of the proposed encoding strategies, we conduct an ablation study in which we remove the signed distance representation (BASD) while preserving the class-aware bounding-box encoding (C-BASD).

It is important to note that class information cannot be removed without fundamentally altering the task definition. The class label specifies which defect type must be generated inside each bounding box, and therefore constitutes a necessary conditioning signal rather than a design choice. For this reason, the only meaningful internal ablation consists of removing the geometric signed distance encoding while keeping the class-aware representation unchanged. This allows us to directly assess the contribution of the boundary-aware signal introduced by BASD.

#### 4.4.1. Impact on Retrieval Ability (EBR)

As shown in Table 5, removing the SDF component degrades the Empty Bounding-Box Rate across most classes. The average EBR increases from 5.51% in the full model to 6.24% without SDF.

The degradation is particularly visible for the crack class (from 2.41% to 3.45%), while other categories remain relatively stable. Although the numerical differences may appear moderate, the consistent increase in miss rate indicates that the signed distance encoding contributes to more reliable activation of the conditioned regions. In other words, BASD improves the robustness of defect retrieval inside the prescribed bounding boxes.

#### 4.4.2. Impact on Spatial Alignment (SAE)

The effect of removing BASD is more pronounced when analyzing spatial alignment. The overall SAE increases from 4.99% to 6.78%, indicating a clear deterioration in boundary consistency. In particular, knot and resin exhibit noticeable increases in misaligned pixels, and crack shows a degradation from 4.57% to 5.61%.

These results suggest that the geometric information encoded by the signed distance map provides a structural prior that guides the denoising trajectory toward spatially coherent defect shapes. Without this boundary-aware signal, the diffusion process still generates defects inside the boxes, but with weaker spatial precision and increased leakage or boundary irregularities.

#### 4.4.3. Controlled Overlap Analysis

To further evaluate the robustness of our encoding in multi-class overlap scenarios, we conduct an additional controlled experiment by generating synthetic samples with predefined bounding-box overlaps of 0.2, 0.3, and 0.4 IoU. We then compute EBR and SAE under these controlled conditions.

As reported in Table 6, the degradation remains limited as overlap increases. Both retrieval reliability (EBR) and spatial alignment (SAE) show only moderate variation across overlap levels, indicating that the proposed deterministic partition strategy provides stable conditioning even in structured intersection regions. These results confirm that the analog bit encoding combined with the geometric overlap partition does not introduce instability in multi-class overlapping configurations.

#### 4.4.4. Discussion

Overall, this ablation confirms that the performance gains observed in Section 4 cannot be attributed solely to the class-enriched encoding. While class information determines what defect to generate, the signed distance representation strongly influences how the defect conforms to its spatial constraint.

The combination of BASD and C-BASD therefore proves essential for achieving both reliable bounding-box retrieval and accurate spatial alignment. In particular, BASD acts as a geometric regularizer that stabilizes conditioning and improves boundary fidelity during the diffusion process.

## 5. Implementation Details

### 5.1. Diffusion Architecture

Our generative model is implemented as a denoising diffusion probabilistic model (DDPM) using a U-Net backbone implemented with the diffusers library (v0.31.0). The network processes inputs of spatial resolution 352×128. The U-Net consists of six downsampling and six upsampling stages with residual blocks (two layers per block) and skip connections. The channel configuration across resolution levels is defined by block_out_channels = [128, 128, 256, 256, 512, 512]. Self-attention is enabled (add_attention = true) and is applied at intermediate resolutions via AttnDownBlock2D and AttnUpBlock2D modules. Group normalization with 32 groups and SiLU activations is used throughout the network. The model takes 10 input channels (in_channels = 10) corresponding to the concatenation of the noisy representation being denoised and the conditioning signals derived from bounding boxes. The denoised representation consists of six channels composed of the RGB image (three channels) and the three-channel analog bit representation of the segmentation masks.

The conditioning signals encode the spatial constraints imposed by the bounding boxes and include:

- a one-channel signed distance map (BASD) capturing the geometric distance to bounding-box boundaries;
- a three-channel analog bit encoding of the bounding-box classes (C-BASD).

These conditioning maps are concatenated with the noisy joint representation, resulting in a total of 10 input channels.

The model predicts 6 output channels (out_channels = 6) corresponding to the denoised joint representation, i.e., the RGB image (3 channels) and the analog bit encoding of the segmentation masks (3 channels).

### 5.2. Noise Schedule and Training Objective

We adopt a linear β noise schedule with $\beta_{\mathrm{start}}=10^{-4}$ and $\beta_{\mathrm{end}}=0.02$ over T=1000 diffusion timesteps. The scheduler is configured with fixed-small variance and ϵ-prediction parameterization. The model is trained to predict the added Gaussian noise using the standard mean squared error objective:

(7) $$L=E_{x,\epsilon ,t}\left[∥\epsilon -\epsilon_{\theta }\left(x_{t},t,c\right){∥}_{2}^{2}\right],$$

where *c* denotes the bounding-box conditioning.

### 5.3. Sampling Procedure

During inference, sampling is performed using the same DDPM scheduler with 1000 reverse-diffusion steps and ancestral sampling. Sample clipping is enabled with range [−1,1], and no dynamic thresholding is applied.

### 5.4. Multi-Class and Overlapping Bounding Boxes

The dataset contains six defect classes. Multi-class conditioning is handled through class-specific encoding maps derived from bounding boxes and class maps. When multiple bounding boxes are present in a single image, their conditioning signals are spatially aggregated at the input level before being processed by the U-Net. Overlapping bounding boxes are resolved implicitly by the convolutional and attention mechanisms during denoising, allowing the model to learn spatial conflict resolution directly from training data.

### 5.5. Training Configuration

The model is trained for 300 epochs using the Adam optimizer with a learning rate of $1\times 10^{-5}$ and a batch size of 8. The random seed is fixed to 1234 for reproducibility. Gradient clipping is applied during training to stabilize optimization. Model checkpoints are saved every 5 epochs and qualitative samples are logged every 10 epochs.

### 5.6. Downstream Segmentation Experiments

For downstream segmentation evaluation, we train separate segmentation models per class to mitigate class imbalance and improve optimization stability. Each model is trained using standard cross-entropy loss on the real and synthetic datasets. Performance is evaluated using Intersection over Union (IoU), F1 scores, and per-class accuracy.

## 6. Conclusions

While synthetic data generation has been explored in various Computer Vision domains, diffusion-based approaches remain limited in jointly generating RGB images and segmentation masks under strong spatial conditioning. In particular, existing layout-conditioned diffusion models struggle to maintain accurate alignment between generated structures and bounding-box constraints, which is critical in industrial defect generation where precise localization is required.

We devised a pipeline to generate synthetic RGB data and its segmentation label counterpart at the same time, starting from bounding-box conditioning. This allows for significantly decreasing the labeling costs while preserving the quality of the segmentation maps.

We validated the performances of our method by comparing our proposal with the current state-of-the art methodology adapted for the setting. We also assessed the quality of our generation through a downstream task, training a UNet with a combination of real and synthetic data.

The experiments suggest that our proposal is robust to spatial consistency generation, improving the performance of the downstream segmentation task.

We also introduced dedicated metrics useful for the community to assess the correctness of layout-conditioned data generation.

## Figures and Tables

**Table 1:** Comparison between our method and [11] in terms of Segmentation Alignment Error. Results are reported as mean ± standard deviation over 3 runs. The Avg is computed over all pixels. Bold values indicate the best performance among competitors; arrows show whether lower (↓) or higher (↑) values are better.

	SAE (%) ↓					
Method	Knot	Crack	Quartzite	Resin	Marrow	Avg
Layout Diffusion [11]	39.45±3.25	84.12±5.17	62.71±4.51	88.77±6.38	55.91±3.81	45.10±4.99
Ours	5.52±0.18	4.56±0.15	3.19±0.10	4.82±0.14	3.65±0.12	5.00±0.16

**Table 2:** Comparison between our method and [11] in terms of Empty Bounding-Box Rate. Results are reported as mean ± standard deviation over 3 runs. The Avg is computed over all bounding boxes. Bold values indicate the best performance among competitors; arrows show whether lower (↓) or higher (↑) values are better.

	EBR (%) ↓					
Method	Knot	Crack	Quartzite	Resin	Marrow	Avg
Layout Diffusion [11]	14.26±2.05	69.00±7.26	48.16±4.18	82.05±5.51	28.79±2.89	25.90±3.29
Ours	0.86±0.04	2.41±0.08	4.99±0.12	2.22±0.07	0.89±0.03	5.52±0.10

**Table 3:** Assessment of generation quality. All metrics are reported as mean ± standard deviation over 3 sampling runs. FID and KID are computed at different InceptionV3 [51] layers, and LPIPS is computed with AlexNet [52], VGG-16 [53], and SqueezeNet [54] backbones. Bold values indicate the best performance among competitors; arrows show whether lower (↓) or higher (↑) values are better.

				**FID ↓**											
**Data**				@2048			@768			@192			@64		
Synth [11]				40.71±1.12			0.26±0.02			24.11±0.90			6.66±0.35		
Synth Ours				45.80±1.25			0.30±0.03			14.57±0.65			3.11±0.18		
				**KID** ↓											
**Data**				@2048			@768			@192			@64		
Synth [11]				40.70±1.10			8.12±0.33			$19\times 10^{3}\pm 0.8\times 10^{3}$			$10\times 10^{3}\pm 0.5\times 10^{3}$		
Synth Ours				45.21±1.25			8.29±0.28			$10\times 10^{3}\pm 0.4\times 10^{3}$			$3.6\times 10^{3}\pm 0.2\times 10^{3}$		
				**LPIPS** ↓											
**Data**				**AlexNet**				**VGG-16**				**SqueezeNet**			
Synth [11]				0.34±0.02				0.50±0.03				0.26±0.02			
Synth Ours				0.27±0.02				0.42±0.03				0.21±0.01			

**Table 4:** Downstream task assessment in terms of F1 score using real, synthetic and real+synthetic data during training. Results are reported as mean ± standard deviation over 3 runs. Bold values indicate the best performance among competitors; arrows show whether lower (↓) or higher (↑) values are better.

	F1 (%) ↑					
Train Data	Knot	Crack	Quartzite	Resin	Marrow	Avg
Real	78.56	48.80	24.49	45.00	65.40	52.45
Synth [11]	72.02±0.41	8.34±0.27	20.91±0.38	18.23±0.31	57.86±0.44	35.47±0.36
Synth Ours	76.41±0.33	45.72±0.48	12.96±0.29	32.55±0.37	58.03±0.42	45.13±0.38
Real+Synth [11]	78.37±0.28	46.84±0.35	26.88±0.41	43.74±0.32	70.92±0.46	53.35±0.31
Real+Synth Ours	79.36±0.30	50.52±0.44	25.71±0.37	46.18±0.39	66.04±0.41	53.56±0.34

**Table 5:** Ablation study of the proposed encoding strategy. Results are reported as mean ± standard deviation over 3 runs. Bold values indicate the best performance among competitors; arrows show whether lower (↓) or higher (↑) values are better.

	**EBR (%) ↓**					
**Method**	**Knot**	**Crack**	**Quartzite**	**Resin**	**Marrow**	**Avg**
BASD + C-BASD	0.86±0.04	2.41±0.08	4.98±0.12	2.22±0.07	0.89±0.03	5.51±0.10
C-BASD	0.96±0.05	3.45±0.11	4.98±0.14	2.23±0.09	0.89±0.04	6.24±0.13
	**SAE (%) ↓**					
**Method**	**Knot**	**Crack**	**Quartzite**	**Resin**	**Marrow**	**Avg**
BASD + C-BASD	5.53±0.18	4.57±0.15	3.19±0.10	4.82±0.14	3.64±0.12	4.99±0.16
C-BASD	6.02±0.20	5.61±0.17	3.34±0.11	6.85±0.22	2.51±0.09	6.78±0.19

**Table 6:** Controlled overlap evaluation with predefined IoU levels. Results are reported as mean ± standard deviation over 3 runs. The limited variation across overlap ratios indicates stable conditioning under multi-class intersections. Arrows show whether lower (↓) or higher (↑) values are better.

	Controlled Overlap (IoU)		
Metric	0.2	0.3	0.4
EBR (%) ↓	5.48±0.12	5.72±0.15	6.01±0.18
SAE (%) ↓	4.87±0.10	5.11±0.13	5.46±0.16

## Data Availability

The data presented in this study are openly available in the GitHub repository at https://github.com/covisionlab/diffusion_labeling (accessed on 10 March 2026).
